# MicroRNAs distribution in different phenotypes of Aortic Stenosis

**DOI:** 10.1038/s41598-018-28246-8

**Published:** 2018-07-02

**Authors:** Iacopo Fabiani, Nicola Riccardo Pugliese, Enrico Calogero, Lorenzo Conte, Maria Chiara Mazzanti, Cristian Scatena, Claudia Scopelliti, Elena Tantillo, Matteo Passiatore, Marco Angelillis, Giuseppe Antonio Naccarato, Rossella Di Stefano, Anna Sonia Petronio, Vitantonio Di Bello

**Affiliations:** 1 Cisanello Hospital AOUP -Department of Surgical, Medical, Molecular Pathology and Critical Area, University of Pisa, Pisa, Italy; 2 Department of Translational Research and new technologies in Medicine and Surgery- University of Pisa, 56100 Pisa, Italy; 3 Fondazione Pisana per la Scienza, 56100 Pisa, Italy

## Abstract

Aortic valve stenosis (AVS) represents a cluster of different phenotypes, considering gradient and flow pattern. Circulating micro RNAs may reflect specific pathophysiological processes and could be useful biomarkers to identify disease. We assessed 80 patients (81, 76.7–84 years; 46, 57.5%females) with severe AVS. We performed bio-humoral evaluation (including circulating miRNA-1, 21, 29, 133) and 2D-echocardiography. Patients were classified according to ACC/AHA groups (D1-D3) and flow-gradient classification, considering normal/low flow, (NF/LF) and normal/high gradient, (NG/HG). Patients with reduced ejection fractionwere characterized by higher levels of miRNA1 (p = 0.003) and miRNA 133 (p = 0.03). LF condition was associated with higher levels of miRNA1 (p = 0.02) and miRNA21 (p = 0.02). Levels of miRNA21 were increased in patients with reduced Global longitudinal strain (p = 0.03). LF-HG and LF-LG showed higher levels of miRNA1 expression (p = 0.005). At one-year follow-up miRNA21 and miRNA29 levels resulted significant independent predictors of reverse remodeling and systolic function increase, respectively. Different phenotypes of AVS may express differential levels and types of miRNAs, which may retain a pathophysiological role in pro-hypertrophic and pro-fibrotic processes.

## Introduction

Aortic valve stenosis (AVS) involvesabout one-fourth of all patient with chronic valvular heart disease^1,2^. It is due to a degenerative calcification process of the aortic cusps and, most commonly, it is a slowly progressive active process, associated with significant Left Ventricular (LV) pressure overload, inducing hypertrophy (LVH) and secondary myocardial fibrosis (MF)^3^. The key findings on transthoracic echocardiogram (TTE) are generally limited to the evaluation of thickening, calcification, reduced systolic opening of the valve leaflets and to flow-dependent parameters assessment (i.e. velocity and gradients), reflecting only the “valvular side” of the pathology^4^. This approach disregards the LV components (function; texture) of the disease, resultingprone to diagnostic discrepancies^5,6^. Recently in literature, in fact, AVS has been depicted as a complex cluster of different phenotypes. Latest classifications are proposing a central role for indexed stroke volume (SVi) (“flow pattern”) to overcome diagnostic inconsistencies^7,8^. Withrespect to this, recent AHA/ACC guidelines for VHD patients management describe 3 distinct phenotypes for symptomatic severe (D) AVS: high gradient (D1); low-flow/low-gradient AVS with reduced Ejection Fraction (EF < 50%), or classical low flow AVS (D2); paradoxical low-flow/low-gradient AVS (D3) (EF > 50%)^9^. Another accredited classification for AVS is based on flow and gradient patterns, identifying 4 groups (group 1: normal flow/low gradient [NF/LG]; group 2: normal flow/high gradient [NF/HG]; group 3: low flow/high gradient [LF/HG]; group 4: low flow/low gradient [LF/LG])^10,11^. Nowadays, cardiac imaging methods and novel biomarkers could provide an integrated assessment of the functional and structural aspects in AVS. In particular, Speckle Tracking Echocardiography (2D-STE) for the evaluation of myocardial deformation represents a more sensible tool to detect cardiac contractile function abnormalitiescompared to traditional parameters (i.e. EF)^12^. Regarding biomarkers, microRNAs (miRNAs) are small non-coding RNAs of about 21-nucleotides that regulate gene expression at a post-transcriptional level. About 2600 miRNAs have been identified in humans, possibly targeting about 30% of human genes^13,14^. Specific targets and biological roles have been assigned to onlyfew miRNAs, although almost every investigated process can be potentially regulated by miRNAs. The expression of many miRNAs shows tissue-specific or stage-specific patterns and their expression is associated with multiple pathological processes (remodeling, hypertrophy, and fibrosis), which have non-negligible effects on the whole cardiovascular system^13,14^. The presence of circulating non-coding RNAs could be related to specific cardiovascular diseases and may represent a useful biomarker for different cardiovascular diseases identification. Considering the pathophysiological implications of AVS on myocardium (hypertrophy) and interstitium (fibrosis, hypertrophy), we selected from literature a pool of microRNAs with an already proved and extensively demonstrated pro-fibrotic (miRNA21) or pro-hypertrophic (miRNA1, 29, 133) modulatory effect at pre-clinical (*in vitro*/tissue) and clinical level (circulating compart) on both myocardium and interstitium^15,16^.

The aim of our study was to evaluate:

- the relative expression of miRNA levels in different phenotypes of severe AVS;
- the association of miRNA levels with structural and functional echocardiographic variables

in a cohort of patients with severe symptomatic AVS evaluated for aortic valve replacement (AVR).

## Materials and Methods

### Study population

Eighty patients with severe symptomatic AVS were prospectively enrolled at theAOUP Cardio-Angiology Unit - Pisaand screened between October 2015 and September 2016. For clinical symptoms, a NYHA Class > II was defined as overt heart failure (HF). Patients underwent laboratory analysis (including miRNA assays), and trans-thoracic echocardiography (standard and 2D-STE). The research was carried out according to the code of ethics of the World Medical Association (Declaration of Helsinki), informed consent was obtained, and the author’s institutional review board (local ethics committee “AziendaOspedalieroUniversitariaPisana”) approved the study. We excluded patients according to the following criteria: age < 18 years old; presence of major comorbidities (i.e. cancer; dialysis treatment; cachexia); not appropriate acoustic window; coronary/ischemic heart disease (including previous acute coronary syndromes or epicardial coronary artery disease, CAD > 50%); pregnancy; moderate or severe valvular disease; inability to sign consent; non-degenerative AVS; diskynetic septum (i.e. pacemaker induced rhythm patients; intraventricular conduction disorders). Follow-up data were collected at 12 months from AVR, using cardiologic visit (outpatient) and echocardiographic exam.

### Conventional Echocardiography

We performed complete transthoracic exams using a heart-dedicated machine (Vivid-E80, General Electric Milwaukee, WI-USA). Lateral decubitus position was the selectedpositionwhile data acquisition was performed with a matrix (M5S) probe at a depth of 16 cm in apical views (two-chamber, four-chamber and apicalviews) and the parasternal view (short - and long-axis views). All parameters were collected according to current recommendations^4,17^. Using standard M-mode/2D images at the parasternal long-axis views we calculated LV dimensions, includingend-diastolic/end-systolic LV diameters, end-diastolic thickness of the interventricular septum and posterior wall. Body surface area (BSA) was utilized to correct left ventricular mass calculation to derive mass index (LVMi). Both the apical 2- and 4-chamber views were considered to evaluate the LV end-diastolic and end-systolic volumes, andstandard Simpson’s rule was utilized to calculate EF. A spectral pulsed-wave Doppler analysis was performed to assess LV diastolic function, measuring early (E wave) and late (A wave) trans-mitral velocities, E/A ratio, and E wave deceleration time (DT). TDI was performed, adjusting gain and frame rate to optimize tissue characterization. The continuity equation was used to calculate the aortic valve area indexed (AVAi), and the modified Bernoulli equation allowedthe estimation of the maximum pressure gradient across the restrictive orifice. Mean trans-aortic pressure gradient (MG) derived from averaging the instantaneous gradients over the ejection period measured by continuous-wave Doppler recordings. We calculated also the valvulo-arterial impedance (Z_VA_) as a measurement of global LV afterload^18^. We used the color Doppler mode optimizing gain and Nyquist limit to evaluate the presence of regurgitant valve disease. The severity of valvular regurgitation was evaluated using a qualitative scale (mild, moderate, and severe), according to the current guidelines^4,17^.

In our study population, we adopted the following classification criteria:

- Reduced EF was defined according to guidelines as EF < 50%^17^;
- Reduced global longitudinal strain (GLS), in accordance with prognostic studies, was defined as a value > −15.9%^19^;
- Concentric Physiology was defined in presence of a Relative Wall Thickness (RWT) > 0.42, while relevant hypertrophy in presence of LVMi > 95 g/m^2^ for women and 115 g/m^2^ for men^20^;
- Low transvalvular gradient was defined in presence of a MG < 40 mmHg^11^;
- Low flow condition was defined in presence of SVi < 35 ml/m^2^ ^7^;
- Abnormal Z_VA_ was considered forvalues > 4.5 mmHg/ml/m^2^ ^18^;
- AHA/ACC classification: D1 (Classical Phenotype: Vmax > 4 m/sec; MG > 40 mmHg; AVAi < 0.6 cm^2^/m^2^), D2 (Classical Low Flow Low Gradient: Vmax < 4 m/sec; MG < 40 mmHg; AVAi < 0.6 cm^2^/m^2^) and D3 (Paradoxical Low Flow Low Gradient: Vmax < 4 m/sec; MG < 40 mmHg; AVAi < 0.6 cm^2^/m^2^, with an SVi < 35 ml/m^2^)^9^;
- Flow-Gradient classification: 1) NF/LG; 2) NF/HG; 3) LF/HG; 4) LF/LG^11^.

### Speckle Tracking Echocardiography

We used 2D-STE(frame rate 45–90 frame/sec, fps) to asses LV GLS, according to current standards^21^. Onlythe global longitudinal component of strain(peak value-mid myocardium)was considered, using a dedicated software (EchoPAC 12, General Electric). To achieve this aim, we acquired standard 2D grey-scale images of the LV at conventional apical 2-,4-chamber and apical long-axis views. By tracking frame-to-frame natural acoustic markers, or speckles, equally distributed within the myocardial wall, 2D-STE represents an angle-independent technology, allowingmyocardial deformation analysis. The percentage change in myocardial length relative to the initial length, according to the strain Lagrangian formula, expresses myocardial strain in percentage. Analysing temporal variation of myocardial strain, we are able to calculate the strain rate that represents a measure of deformation rate. The longitudinal deformation consists in motion from mitral annulus to the apex in the apical views and may be negative (shortening) or positive (lengthening). We traced manually the endocardial contour at the end-systolic frame. Thenthe software, automatically, traced a concentric region of interest (ROI), including the entire myocardial wall. We verified the myocardial tracking, and we adjusted the ROI to optimize the tracking, where needed. Dividing each LV image into 6 segments, we performed a global and segmental strain analysis. A random sample of 10 patients was re-analysed by 2 independent observers. The intraclass correlation coefficient (ICC) and its 95% confidence intervals (CIs) were calculated to evaluate inter- and intra-observer reproducibility of 2D-STE. We obtained a good intra-observer and inter-observer repeatability (ICCs > 0.80).

### Invasive Measurements

Standard left heart catheterization was performed before AVR in 60 patients, in addition to coronary angiography. Invasive end-diastolic pressure (EDPi; fluid-filled catheter), peak-to-peak gradient, and semi-quantitative aortic regurgitation were evaluated.

### Circulating miRNAs Study

We collected peripheral blood samples in specific tubes (PAXgene Blood RNA Tube), designed for immediate stabilization of peripheral blood RNA (PreAnalytiX). Blood samples were collected and frozen at −80 °C within 2 hours after blood test. Frozen samples were equilibrated at room temperature for 2 hours before processing. RNA was isolated using the PreAnalytiXPAXgene miRNA isolation kit according to the manufacturer’s protocol. We estimated the quantity of RNAusing 2 µl of undiluted RNA solution anda Qubit 2.0 Fluorometer (Life Technologies). The resultsranged from 50 to 500 ng/µl of RNA. miRNAs were reverse transcribed from 500 ng of total extracted RNA sample using the miScript II RT Kit (QIAGEN). miRNA expression analysis was performed three times using 1 µl of cDNA as a template for real-time PCR with the miScript SYBR Green PCR Kit (QIAGEN) and the miScript Primer Assays (SNORD96 - assay code MS00033733, miRNA21 – assay code MS00009079, miRNA1 – MS00008358, miRNA133 – MS00031423, miRNA29 – MS00003269 assay code (QIAGEN)) according to manufacturer’s instructions on the CFX96 Real Time system c1000 thermal cycler (BIORAD). Data analysis was performed using the Bio-Rad CFX Manager Software v3.1. miRNA21, miRNA1, miRNA133 and miRNA29 expression was calculated using SNORD96 expression level as reference and the relative normalized expression ∆∆Cq formula. The number of technical replicates used for miRNA21, miRNA1, miRNA133 and miRNA29 was 3. We reported normalized expression levels respective to a housekeeping gene.

All miRNA expression analyses were adjusted for estimated glomerular filtration rate (eGFR) values.

In 38 subjects (47.5%) (*see Supplementary Material and Table* for details), in order to avoid the potential confounder effect, among others, of circulating blood cells, limiting cardiac specificity of miRNAs here tested, we validated circulating whole blood vs plasmatic compart by mean of linear regression analysis, also calculating inter-rater correlation coefficients (ICC). In the present paper we did not report a correlation between miRNAs expression levels and circulating blood cells.

### Statistical Analysis

The Kolmogorov–Smirnov test for normality was used to assess the data. Continuous variables are presented as mean and 95% confidence intervals if normally distributed or median and range interquartile if not normally distributed. Percentage was used for categorical data. We treated the circulating miRNA levels as continuous variables, or we normalized them with Log transformation. Continuous variables were compared using Student’s t-test or Mann-Whitney U test when non-Gaussian. Correlation r coefficient (or Spearman’s rho) where assessed as appropriate. ANOVA or Kruskal-Wallis test were used to evaluate distribution of miRNA levels in different classification categories, with appropriate post-hoc corrections for interactions. For discrimination of patients with reduced SVi we used a c-statistic approach to derive the miRNA value with the best combination of sensitivity and specificity. Multivariate, stepwise regression analysis was used to identify predictors of reverse remodeling (Model 1) and systolic function recovery (Model 2). In the text we considered GLS in absolute value in order to preserve the statistical meaning of regression analysis (direct/inverse correlation/association). The threshold for statistical significance was p < 0.05. The following statistic package was used: Medcalc 12.7 (Medcalc Software 2013, Belgium).

## Results

Table 1 summarizes clinical characteristics of our patient population (80 subjects with a confirmed diagnosis of severe AVS), and in Table 2 all the common echocardiographic variables describing AVS are indicated.

**Table 1:** Clinical characteristics of study population (n = 80), risk factors and comorbidities.

Gender (Male)	34	42%
Age (years)	81	76.7–84.0
Weight (Kg)	73	69.8–75.3
Height (cm)	164	164.2–167.9
BMI (kg/m^2^)	26	25.5–27.1
BSA (m^2^)	1.8	1.78–1.86
SAP (mmHg)	137.6	133–141
DAP (mmHg)	71.5	68–74
Smokers	11	13.6%
Family history of cerebro-vascular disease	18	22.2%
Diabetes Mellitus	21	25.9%
Hypertension	70	86.4%
Dyslipidaemia	11	13.6%
Obesity	16	19.7%
History of Angina	24	29.6%
CAD (<50% Epicardial Vessels)	35	43.2%
COPD	18	22.2%
NYHA class I/II	48	59.3%
NYHA class >II	33	40.7%
Syncope	9	11.1%
Atrial Fibrillation	9	11.1%
eGFR (mL/min/1.73 m^2^)	58.667	46.4–76.1
CKD	46	56.8%
Anaemia	18	22.2%
White Blood Cells (103/µL)	8	5–10.5
Erythrocytes (106/µL)	4	3.5–6
Platelets (103/µL)	180	140–230
EuroSCORE II (%)	2.6	1.7–4.0
Logistic EuroSCORE (%)	8.38	4.7–15.1

Note: the data are presented as number and %, mean and 95% confidence intervals if normally distributed or median and range interquartile if not normally distributed.

BMI: body mass index; BSA: body surface area; CAD: coronary artery disease; CKD: chronic kidney disease; COPD: chronic obstructive pulmonary disease; DAP: diastolic arterial pressure; eGFR: estimated glomerular filtration rate; NYHA: New York Heart Association; SAP: systolic arterial pressure.

**Table 2:** Echocardiographicparameters.

LV EDVi (ml/m^2^)	48	40.6–58.6
LV ESVi (ml/m^2^)	24	16.9–33.0
IVS (mm)	13	12–14
RWT	0.508	0.488–0.527
LAVi (cm^2^/m^2^)	44	40.6–47.3
E/A	0.729	0.55–1.05
DT (ms)	232	215.2–248.4
E/e′(average sept + lat/2)	17	15.1–18.3
s′ septal (cm/s)	6	5.3–6.0
s′ lateral (cm/s)	6	5.0–7.4
EF (%)	61	54.7–68.1
GLS (%)	−14	13.2–14.7
LVMi (g/m²)	134	127.3–139.9
LVH	73	91.2%
EstimatedsPAP	30	25–35
Systolicdysfunction (EF < 50%)	14	17%
Diastolic dysfunction (grade I/II/III)	13/47/20	16.3/58.7/25%
Stroke Volume index (ml/m^2^)	35	33.1–37.7
AVAi (ml/mq)	0.359	0.33–0.38
AS jet velocity (m/s)	4	4.29–4.50
Maximum pressure gradient (mmHg)	77	69.0–87.8
Mean pressure gradient (mmHg)	47	41.0–53.8
Velocity Ratio	0.202	0.19–0.21
ZVA (mmHg/mL/m^2^)	5	4.280–6.06
TAPSE (mm)	20	17.75–24
ACC/AHA GROUP: D1/D2/D3	62/14/4	77.5%/17.5%/5%
FLOW/GRADIENT GROUP: NF-LG/NF-HG/LF-HG/LF-LG	17/32/13/4	21%/40%/16%/4%

Note: the data are presented as number and %, mean and 95% confidence intervals if normally distributed or median and range interquartile if not normally distributed. ACC/AHA: American College of Cardiology/American Heart Association group: D1) Classical Phenotype (Vmax > 4 m/sec; MG > 40 mmHg; AVAi < 0.6 cm^2^/m^2^); D2) Classical Low Flow Low Gradient (Vmax < 4 m/sec; MG < 40 mmHg; AVAi < 0.6 cm^2^/m^2^); D3) Paradoxical Low Flow Low Gradient (Vmax < 4 m/sec; MG < 40 mmHg; AVAi < 0.6 cm^2^/m^2^, with an SVi < 35 ml/m^2^). FLOW/GRADIENT GROUP: NF normal flow (Svi > 35 ml/m^2^); LF low flow (SVi < 35 ml/m^2^); LG low gradient (mean pressure gradient < 40 mmHg); HG high gradient (mean pressure gradient > 40 mmHg).

AS: aortic stenosis; AVAi: aortic valve area index; DT: deceleration time; E/A: ratio of early to late diastolic mitral filing velocity (PW); EDVi: indexed end-diastolic volume; E/e′: ratio of trans-mitral early diastolic velocity (PW) to tissue early diastolic mitral annular velocity (TDI); EF: ejection fraction; ESVi: end-systolic volume indexed; GLS: global longitudinal strain; IVS: inter-ventricular septum; LAVi: indexed left atrium volume; LVMi: indexed left ventricular mass; LV: left ventricle; LVH: left ventricular hypertrophy (defined as LVMi >115 g/m^2^ in males or >95 g/m^2^ in females); sPAP systolic pulmonary artery pressure; RWT: relative wall thickness; TAPSE: tricuspid annular plane systolic excursion; ZVA: valvulo-arterial impedance.

In Table 3, normalized expression levels of circulating miRNAs (miRNA1-21-29-133) are specified. Principal characteristics of population are reported in Table 4 according to the ACC/AHA and Flow/Gradient classification (preserved EF).

**Table 3:** Relative expression levels of circulating miRNAs.

microRNA	Normalized Expression Levels	95%CI	Number of Experimental Replicates
miRNA1	0.262	0.0733–1.399	3
miRNA133	1.324	0.637–10.236	3
miRNA29	1.256	0.535–8.042	3
miRNA21	2.471	0.867–8.012	3

Note: the data are presented as number, mean and 95% confidence intervals if normally distributed or median and range interquartile if not normally distributed.

**Table 4:** Distribution of the principal variables according to ACC/AHA and Flow/Gradient classification.

Variable	Flow/Gradient Groups					Acc/Aha Groups			
	NF/LG (17)	NF/HG (32)	LF/HG (13)	LF/LG (4)	p	D1 (62)	D2 (14)	D3 (4)	p
Gender (% of female)	43%	50%	87%	33%	**0.01**	63%	29%	67%	**0.05**
E/e′ average	15.6 (9.2–22)	15.7 (14–18)	18.2 (14.5–22)	19.3 (16.5–22)	ns	15.3 (15.6–19)	15.6 (12.3–18.4)	17 (15.2–18)	ns
AGE, years	78 (67–88)	78 (74–81)	80 (78–82)	81 (79–84)	ns	79.4 (77.5–81)	78.1 (72.3–84)	79.3 (73–87)	ns
EDVi, ml/m^2^	52 (44.5–59)	52 (48–56)	46 (43–48.7)	45 (41–48)	**0.01**	49.2 (46–52.3)	70 (53–84)	48.2 (41–64)	**<0.0001**
EF%	62 (54–71)	68 (65–70)	63 (61–66)	60 (55–66)	ns	63 (59.5–65.2)	49 (40.7–53.1)	60 (57.5–61.3)	0.01
GLS%	−16.3 (13.8–17)	−15.3 (14–16.4)	−14.4 (12.9–15.9)	−11.5 (10–15.5)	ns	−14.5 (13.6–15)	−10 (9.5–12.3)	−13 (10.7–14.7)	0.05
LAVi, ml/m^2^	41.4 (31.8–55)	43.7 (38.1–49)	46 (38.2–52)	47 (41–55)	ns	45 (41–48.7)	43 (36–49)	35 (28.2–38)	ns
LVMi, g/m^2^	131 (94–160)	127 (116–137)	127 (118–135)	133 (114–143)	ns	132 (125–138)	145 (127–155)	136 (89–155)	ns
RWT	0.5 (0.44–0.57)	0.5 (0.45–0.55)	0.54 (0.50–0.57)	0.55 (0.51–0.57)	ns	0.51 (0.5–0.54)	0.44 (0.4–0.5)	0.54 (0.46–0.58)	**0.005**
ZVA, mmHg/ml/m^2^	4.4 (3.4–5.3)	4.6 (4–5.2)	5.5 (5.1–5.8)	6.1 (5.5–6.3)	**0.01**	5.2 (4.8–5.5)	4.8 (4.2–5.5)	5.5 (4.6–6)	ns

Note: the data are presented as number and %, mean and 95% confidence intervals if normally distributed or median and range interquartile if not normally distributed. ACC/AHA: American College of Cardiology/American Heart Association group: D1) Classical Phenotype (Vmax > 4 m/sec; MG > 40 mmHg; AVAi < 0.6 cm^2^/m^2^); D2) Classical Low Flow Low Gradient (Vmax < 4 m/sec; MG < 40 mmHg; AVAi < 0.6 cm^2^/m^2^); D3) Paradoxical Low Flow Low Gradient (Vmax < 4 m/sec; MG < 40 mmHg; AVAi < 0.6 cm^2^/m^2^, with an SVi < 35 ml/m^2^). FLOW/GRADIENT GROUP: NF normal flow (Svi > 35 ml/m^2^); LF low flow (SVi < 35 ml/m^2^); LG low gradient (mean pressure gradient < 40 mmHg); HG high gradient (mean pressure gradient > 40 mmHg).

EDVi: indexed end-diastolic volume; E/e′: ratio of trans-mitral early diastolic velocity (PW) to tissue early diastolic mitral annular velocity (TDI); EF: ejection fraction; GLS: global longitudinal strain; LAVi: indexed left atrium volume; LVMi: indexed left ventricular mass; RWT: relative wall thickness; ZVA: valvulo-arterial impedance.

**Figure 1:**
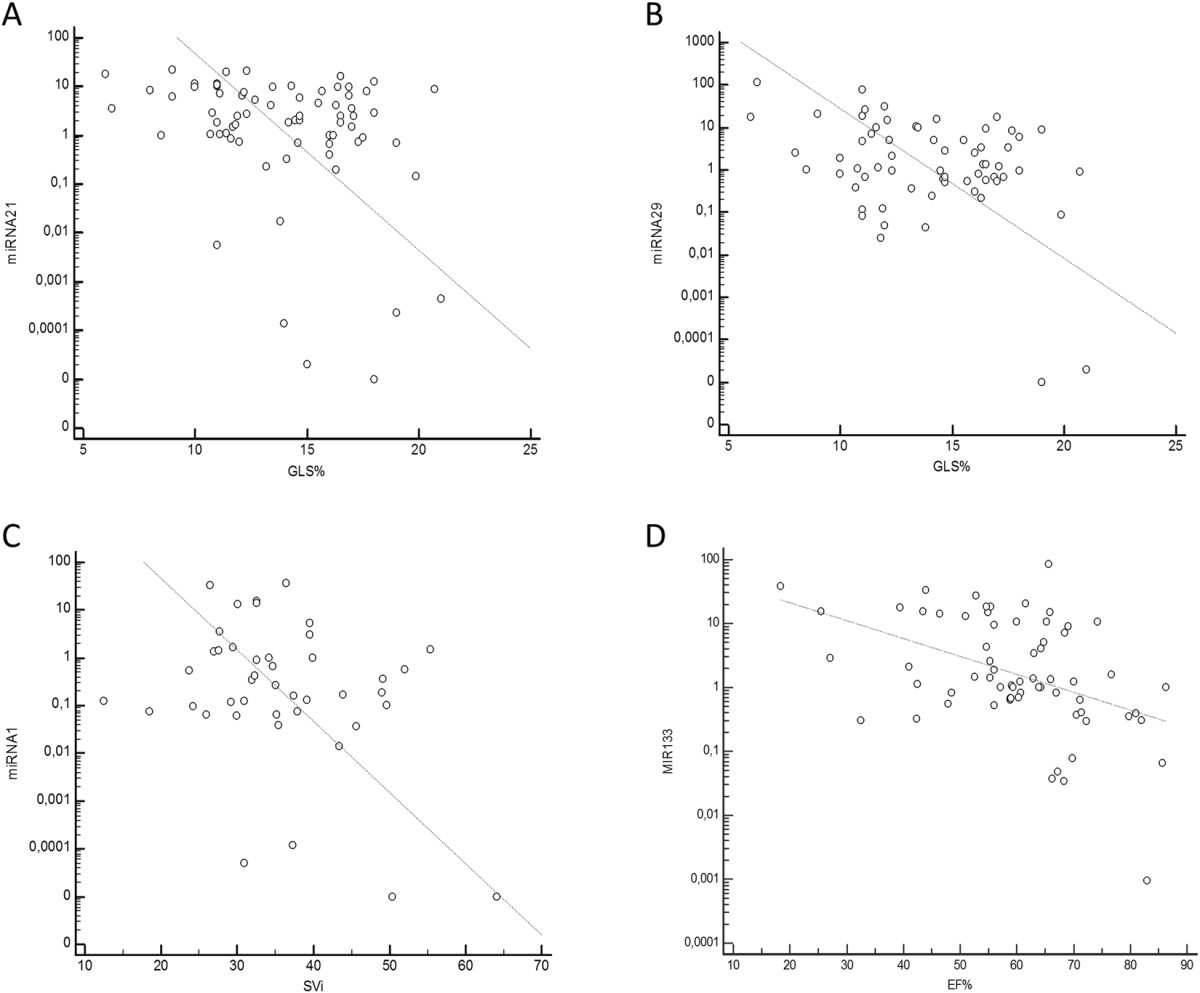
Correlation analysis. (**A**) Correlation between global longitudinal strain (GLS, % in absolute value) and microRNA21 (miRNA21, Log Scale): r = −0.3, p = 0.0009. (**B)** Correlation between GLS and microRNA29 (miRNA29, Log Scale): r = −0.38; p = 0.001. (**C**) Correlation between indexed stroke volume (SVi, ml/m^2^) and micro RNA1 (miRNA1, Log Scale): r = −0.32; p = 0.003. (**D**) Correlation between ejection fraction (EF%) and micro RNA133 (miRNA133, Log Scale): r = −0.45; p = 0.002.

**Figure 2:**
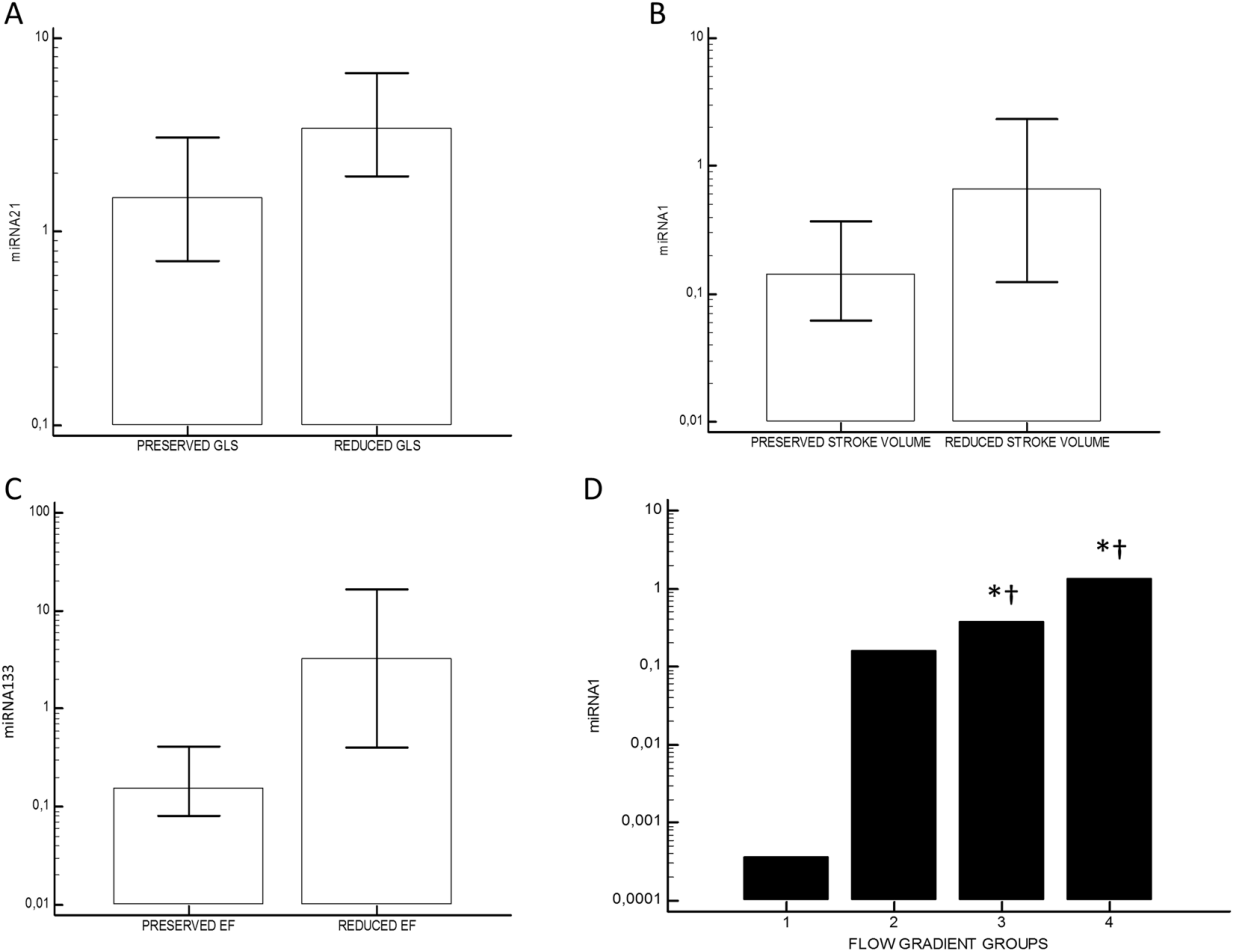
Differential distribution of miRNA expression levels. (**A**) miRNA21 in patients with reduced vs preserved global longitudinal strain (GLS, cut-off: −15.9%): 3.45, 1.94–6.6 vs 1.5, 0.71–3.08; p = 0.031. (**B**) miRNA1 in patients with reduced vs preserved stroke volume indexed (cut-off: 35 ml/min): 0.67, 0.12–2.48 vs 0.14, 0.06–0.37; p = 0.02. (**C**) miRNA133 in patients with reduced vs preserved ejection fraction (EF, cut-off: 50%): 7.9, 1.01–16.29 vs 1.03, 0.76–1.68; p = 0.03. (**D**) miRNA1 distribution according to flow-gradient classification (Group 1: Normal Flow/Low Gradient; Group 2: Normal Flow/High Gradient; Group 3: Low Flow/High Gradient; Group 4: Low Flow/Low Gradient). *p < 0.05 in comparison with group 1; ^†^p < 0.05 in comparison with group 2.

We observed 14% of patients (n = 11) with reduced EF. Circulating miRNA21 showed inverse correlation with GLS in absolute value (r = −0.3, p = 0.009) and a direct correlation with E/e′ (r = 0.33, p = 0.058; Fig. 1A); miRNA29 had inverse correlation with age (r = −0.34, p = 0.004), eGFR (r = −0.37, p = 0.02) and GLS (r = −0.38, p = 0.001; Fig. 1B); miRNA1 demonstrated inverse correlation with SVi (r = −0.32, p = 0.03; Fig. 1C) while miRNA133 inverse correlation with DT(r = −0.39, p = 0.001), EF (r = −0.45, p = 0.002; Fig. 1D), eGFR (r = −0.3, p = 0.01), age (r = −0.3, p = 0.01), and direct correlation with E/A ratio (r = 0.36, p = 0.03), ESV (r = 0.44, p = 0.002), sPAP (r = 0.37, 0.002), EDPi (r = 0.37, p = 0.05). Using a c-Statistic approach, miRNA1 had a Sensitivity of 77% and a Specificity of 68% (AUC 0.7; p = 0.01) for discriminating reduced SVi subjects. Levels of miRNA21 were increased in patients with a diagnosis of overt HF (6.68, 2.83–9.81 vs 1.85, 1.01–2.59; p = 0.01) and reduced GLS (3.45, 1.94–6.6 vs 1.5, 0.71–3.08; p = 0.03; Fig. 2A). A reduced SVi was characterized by higher levels of miRNA1 (0.67, 0.12–2.48 vs 0.14, 0.06–0.37; p = 0.02; Fig. 2B) and miRNA21 (4.16, 1.98–7.99 vs 1.72, 0.7–2.85; p = 0.02). Patients with reduced EF were characterized by significantly higher levels of miRNA1 (0.16, 0.08–0.41 vs 3.3, 0.5–18.02; p = 0.003; Fig. 2C) and miRNA133 (7.9, 1.01–16.29 vs 1.03, 0.76–1.68; p = 0.03). The presence of significant LVH was associated to higher levels of miRNA133 (1.51, 1.01–4.86, versus 0.64, 0.31–1.33; p = 0.02). Distribution of levels of miRNA29 was significantly different when we considered the 3 sub-classes of symptomatic severe AVS (D stage: D1, classical phenotype, 77%, D2, low/flow low gradient with reduced EF, 17%; D3, paradoxical low flow/low gradient 4%; p = 0.03, with higher levels for D2). Considering the flow/gradient classification of AS, we observed 14 patients (21.2%) with normal SVi/low MG; 32 (37.5%) withnormal SVi/high MG; 13 (16.2%) with low SVi/high MG; 4 (3.8%) with low SVi/low MG. OnlymiRNA1 showed a significant different distribution in the groups, with higher values for group 3 and 4 (Overall p for ANOVA = 0.005; 1 vs 2 p = ns; 3 vs 2 p = 0.05; 3 vs 1 p = 0.001; 3 vs 4 p = ns; 4 vs 1 p < 0.0001; 4 vs 2 p = 0.005 Fig. 2D). There were no age-related, gender, or ethnic differences in study population.

### Follow-up

Follow-up data were available in 75 subjects (94% of cases). Thirty patients underwent surgical valve replacement (37.5%, of which 25 [83%] with a biologic prosthesis), while 50 (62.5%) underwent percutaneous procedure. After an average follow-up of 15 ± 3 months, no major procedural complications nor long-term cardio-vascular events were noted. An increase in AVAi was observed (baseline AVAi 0.36 cm^2^, 95% CI 0.33–0.38 vs follow-up 1.5 cm^2^, 95% CI 1.36–1.6; p < 0.0001; Delta AVAi 1.02, 95% CI 0.93–1.11 cm^2^), as well as a reduction in peak trans-valvular velocity (baseline Vmax 4 m/sec, 95% CI 4.3–4.5 vs follow-up 2.1 m/sec, 95% CI 2–2.28; p < 0.0001; Delta V max 2.12, 95% CI 2.01–2.35 m/sec). A significant reverse remodeling was also observed (baseline LVMi 134 g/m^2^, 95% CI 127.3–140 vs follow-up 119.6, 95% CI 114.6–124.5; p < 0.0001; Delta LV mass indexed 16.07, 95% CI 6.77–25.38 g/m^2^). No significant increase in EF was reported (baseline EF% 61, 95% CI 54.7–68.1 vs follow-up 62, 95% CI 55.5–69; p ns; Delta EF 2.34, 95% CI −1.45–6.17%), while GLS significantly improved from baseline (baseline GLS% −14, 95% CI 13.2–14.7 vs Follow-up −15.6, 95% CI 14.7–16.4; p = 0.01; Delta GLS in absolute value 0.52, 95% CI 0.21–0.72%). A significant correlation between Delta AVAi and Delta GLS was found (r = 0.33; p = 0.01). In a multivariate stepwise regression model (Table 5a), using reverse remodeling (LV Mass Reduction) as a dependent variable, miRNA21 levels resulted significant independent predictors with baseline eGFR and Delta Vmax (R^2^ = 0.89), while, in another model (Table 5b), miRNA29 baseline levels and Delta AVAi emerged as independent predictors of Delta GLS (R^2^ = 0.58). No significant gender differences were monitored during follow-up data.

**Table 5:** Delta LV mass and Delta GLS Multivariate regression model.

Independent variables	Coefficient	Std. Error	r_partial_	t	p
**a**. **Regression model: Delta LV Mass**					
(Constant)	376.6904				
eGFR (mL/min/1.73 m^2^)	0.3180	0.1435	−0.5095	−2.216	0.0438
miRNA21	−4.6870	0.9933	−0.7835	−4.718	0.0003
Delta V max (m/sec)	75.3057	6.9720	−0.9449	−10.801	< 0.0001
**b**. **Regression model: Delta GLS**					
(Constant)	−0.002970				
miRNA29	−0.07732	0.01347	0.7606	5.739	< 0.0001
Delta AVAi (cm^2^/m^2^)	0.082	0.01647	0.6606	5.49	< 0.0001

Note: Delta GLS was considered in absolute value.

AVAi: aortic valve area indexed; eGFR: estimated glomerular filtration rate; GLS: global longitudinal strain; LV: left ventricle.

## Discussion

Principal findings of our study are

- miRNAs have a differential distribution considering different phenotypes of severe AVS: a low flow condition is associated with increased levels of miRNA21, miRNA1 and miRNA133, while LVH is reflected by higher levels of miRNA133;
- miRNA21 levels reflect both functional and hemodynamic impairment;
- a more advanced stage of AVS, typically characterized by a reduced EF, is associated with higher levels of miRNA29, miRNA133 and miRNA1;
- miRNA21 and miRNA29 result independent predictors of reverse remodelling and systolic function recovery after aortic valve replacement.

Severe AVS is associated with increased hemodynamic load and significant LV remodeling (encompassing hypertrophy and fibrosis), finally leading to impaired myocardial function and poor outcome in the absence of appropriate treatment^1,3^. However, as previously stated, the main focus of routine evaluation of AVS is the assessment of valvularparameters, with a limited role attributed to LV compartment^2,4^. Moreover, discrepancies are frequently observed between the parameters, especially when a low-flow state exists as a result of reduced EF or in case of severe LV remodeling despite preserved EF (>50%)^5,10^. Several papers recently reported the determinant prognostic role of SVi, as a strong indicator of functional and flow conditions: reduced SVi is associated with advanced ventricular remodeling condition and a poor outcome^6,10,22,23^. Furthermore, LV response in terms of hypertrophy and fibrosis is peculiar in patients with AVS, because several factors (including age, sex, Z_VA_, bio-modulators)play different roles at different levels and in different stages of the disease^18,24^. Current knowledge allows us to assert that miRNAs might represent important regulators of valvular disease development. Available data suggest that distinct miRNAs are dysregulated in AVS, supporting different underlying pathophysiological mechanisms. miRNA21 is up-regulated in cardiomyocytes during the fibrotic process; indeed, both myocardial and circulating levels of miRNA21 are significantly higher in patients with AVS compared to controls, correlating with MG and fibrosis. Previous data from our group also showed how miRNA21 manifested both in interstitial tissueand myocytes, with the fibrous tissue representing the site of major expression^25–27^. In this study, miRNA21 levels are associated with an impaired myocardial deformation, evaluated by GLS, and a reduced SVi, together with altered diastolic function and increased non-invasively-estimated filling pressure. Both miRNA29 and miRNA133 are modulators of fibrotic and hypertrophic processes and may reflect these 2 coexisting conditions also in AVS, potentially identifying an advanced or rapidly progressing valvular disease. In details, miRNA29 family is down-regulated in fibroblasts during myocardial fibrosis while it is up-regulated in myocardial hypertrophy; miRNA133 is down-regulated in cardiomyocytes during myocardial fibrosis and hypertrophy development. Combiningthe analysis of these modulators together with clinical parameters, it has been possible to predict LV mass normalization at one year after AVR^28–30^. Indeed, in our study miRNA29 and miRNA133 levels were significantly associated with some of the principal pro-fibrotic conditions (e.g. inverse correlation with eGFR and age), leading to impaired myocardial deformation and increased filling pressures. Interestingly, miRNA29 showed a differential distribution according to ACC/AHA stages of Valvular AS, with significantly higher levels in D2 patients (i.e. low-flow/low-gradient). Previous findingsdemonstratedboth LVH and advanced systolic compromise (namely EF reduction) represent relevant triggers for miRNA133 release^31^. We confirmed these data in our study, showing miRNA133 levels significantly increased in patients with reduced EF and advanced LVH. We focusedalso on miRNA1, described as down-regulated in cardiomyocytes during myocardial hypertrophic process^32,33^. In our population, miRNA1 levels increased in presence of reduced SVi and EF, with a significant differential distribution in 4-group flow-gradient classification (with higher levels in LF/HG and LF/LG groups). These findings further emphasize the link between the hypertrophic response and the impaired LV flow and function, proposing miRNA1 as a reliable index of flow condition and ventricular function, especially in AVS. Finally, according to literature, we reported a reduction of LV mass during follow-up (reverse remodeling) and an increase in longitudinal systolic function (GLS)^12,34^. Interestingly, in our regression models, baseline miRNA 21 and 29 levels resulted predictors of mass reduction, as previously shown^30^, and systolic function increase, assessed in terms of GLS, respectively. Then, we can speculate that the available evidence baseline miRNA levels may be helpful diagnostic tools in terms of prediction of post-operative LV remodeling.

## Limitations

Our population, although significant, could result too limited in number since the multiple phenotypes of AS patients described. Furthermore, a control population for miRNAs was not included. However, previous papers hadalready shown data on AVS patients vs a normal population, so we mainly aimed at examining a differential miRNA-level distribution in anhomogeneous population (severe symptomatic AVS).

## Conclusions

AVS Common characterization cannot fully elucidate the complex pathophysiology of the cardiac remodeling process. Myocardial hypertrophy, fibrosis and apoptosis represent the main actors of cardiac remodeling in AVS: these patterns are regulated not only by pressure overload, but also by a complex network of humoral modulators. Our study aims at proposing a model of translational research inAVSfor a better diagnostic and prognostic patient stratification. Different phenotypes of AVS, with variable degrees of functional and hemodynamic impairment, express differential levels and types of circulating miRNAs. In this scenario, miRNAs could supply a more integrated knowledge of the disease and become amarker of fibrosis/adverse hypertrophic stimulus^34^, even predicting the development of systo-diastolic dysfunction.

## Electronic supplementary material

SUPPLEMENTARY MATERIAL
