# Supplementary material for: MicroRNAs distribution in different phenotypes of Aortic Stenosis
Source: Sci Rep. 2018 Jul 2;8:9953. doi: 10.1038/s41598-018-28246-8 (PMC6028376; doi:10.1038/s41598-018-28246-8)
Supplement: Supplementary file 1 — SUPPLEMENTARY MATERIAL [file 41598_2018_28246_MOESM1_ESM.pdf]

"MicroRNAs distribution in different phenotypes of Aortic Stenosis"

Running Title: *MicroRNAs in Different Phenotypes of Aortic Valve Stenosis.*

Authors

Fabiani Iacopo, MD[1§], Pugliese Nicola Riccardo, MD[1§], Calogero Enrico, MD[1],Conte Lorenzo, MD[1], Mazzanti Chiara Maria, PhD[3], Scatena Cristian, MD[2], Scopelliti Claudia, PhD[3], Tantillo Elena, PhD[3], Passiatore Matteo, MD[2], Angelillis Marco, MD[1], Naccarato Antonio Giuseppe, MD[2], Di Stefano Rossella, MD[1], Petronio Anna Sonia, MD[1] and Di Bello Vitantonio, MD[1†]*

1)Cisanello Hospital AOUP - Department of Surgical, Medical, Molecular Pathology and Critical Area, University of Pisa- Pisa, Italy **2)** Department of Translational Research and new technologies in Medicine and Surgery - University of Pisa-56100-Pisa, Italy. **3)** Fondazione Pisana per la Scienza-56100-Pisa, Italy.

§The first two authors contributed equally to this work and are joint first authors

Supplementary Material

Plasmatic and Whole Blood miRNAs evaluation

In 38 (47.5%) patients, a 5-ml sample of peripheral blood (PB) in EDTA-containing Vacutainer tubes, drawn at the same time of PAXgene Tubes, was stored at -80°C on ice. The PB samples were processed within four hours of draw. All centrifugation steps were performed at 4°C. Samples were initially spun at 1900 x g (3000 rpm) for 10 minutes to separate the plasma from the Buffy Coat and Red Blood Cells. Careful transfer in a new tube (RNAsy-free) of the upper phase, without disturbing the intermediate buffy coat layer (containing white blood cells and platelets), was done. The plasma was then re-centrifuged at 16000 x g for 10 minutes to remove additional cellular nucleic acids attached to cell debris. Total RNA was extracted with miRNeasy Mini Kit, following the manufacturer's instructions. Each RNA sample was quantified with a spectrophotometer (NanoDrop 1000, Thermo Scientific, Wilmington, Delaware). The results ranged from 50 to 500 ng/µl of RNA. miRNAs were reverse transcribed from 500 ng of total extracted RNA sample using the miScript II RT Kit (QIAGEN) and qRT-PCR was conducted as described in the *Methods (Circulating miRNAs Study)* section of the Manuscript. Relative Expression (fold-change) was calculated using SNORD96 expression level as reference and the relative normalized expression ΔΔCq formula.

Then, we made a linear regression analysis with whole blood (Supplementary Table 1). To confirm the reliability of measurements, we calculated inter-rater correlation coefficient (ICC) in terms of consistency, obtaining a good inter-measurement reproducibility for all the miRNA samples.

Supplementary Table 1. Linear regression analysis and inter-rater correlation coefficient (ICC) of miRNA relative expression levels measured in plasma and whole blood (see in main manuscript Table 3 for miRNA expression levels in the whole blood).

**Plasmatic miRNA 1 (0.001, 95% CI 0.0001–0.03)***	
R^2	0.81
Constant	17.4
Coefficient	253
P	<0.001
ICC	0.85 (0.78-0.89)
**Plasmatic miRNA 21 (4.06, 95% CI 0.84 – 13.2)***	
R^2	0.77
Constant	42.8
Coefficient	327
P	<0.001
ICC	0.82 (0.74-0.85)
**Plasmatic miRNA 29 (3.55, 95% CI 0.69 – 11.93)***	
R^2	0.82
Constant	35.9
Coefficient	354
P	<0.001
ICC	0.86 (0.77-0.90)
**Plasmatic miRNA 133 (8.61, 95% CI 0.56 – 21.61)***	
R^2	0.85
Constant	23.2
Coefficient	287
P	<0.001
ICC	0.88 (0.81-0.92)

*indicates plasma miRNA expression levels with 95% Confidence Interval (CI)
